# Comparison of Intravitreal Aflibercept and Ranibizumab following Initial Treatment with Ranibizumab in Persistent Diabetic Macular Edema

**DOI:** 10.1155/2018/4171628

**Published:** 2018-04-19

**Authors:** Ali Demircan, Zeynep Alkin, Ceren Yesilkaya, Gokhan Demir, Burcu Kemer

**Affiliations:** Beyoglu Eye Research and Training Hospital, Istanbul, Turkey

## Abstract

**Purpose:**

To compare the visual and anatomic outcomes in patients with persistent diabetic macular edema (DME) who switched from ranibizumab to aflibercept with those who continued with previous ranibizumab therapy.

**Methods:**

In this retrospective comparative study, medical records of consecutive patients with center-involved DME ≥ 350 *μ*m who had at least three recent consecutive monthly ranibizumab injections followed by as-needed therapy with either aflibercept or ranibizumab were reviewed. Data were collected at presentation (preinjection), at the intermediary visit, and at the last visit (at the end of the follow-up period).

**Results:**

Forty-three eyes of 43 patients were divided into two groups: the switch group (*n* = 20) and the ranibizumab group (*n* = 23). Though no significant improvement was found in the mean BCVA from the intermediary visit to the last visit, there was a difference in the mean CMT in the switch group and the ranibizumab group (*p* < 0.001 and *p* = 0.03, resp.). The mean CMT decreased after the intermediary visit by 188.6 ± 120.5 *μ*m in the switch group and by 60.3 ± 117.1 *μ*m in the ranibizumab group (*p* = 0.003).

**Conclusions:**

Both aflibercept and ranibizumab decreased CMT in patients with persistent DME who showed a poor response to ranibizumab injections. However, switching to aflibercept provided only morphologic improvement.

## 1. Introduction

Diabetic retinopathy is the leading cause of visual impairment among working-age people aged <45 years around the world and is rising in prevalence [1, 2]. Diabetic macular edema (DME) leads to visual impairment in diabetic retinopathy, and its prevalence has been estimated as 6.8% in the diabetic population [3]. Currently, clinical trials providing level 1 evidence have revealed that antivascular endothelial growth factor (VEGF) agents, United States Food and Drug Administration-approved ranibizumab and aflibercept, as well as off-label bevacizumab, are the most effective treatment options for improvement of visual acuity and macular morphology for center-involving DME compared with laser [4–6]. The RISE-RIDE trials for ranibizumab, VIVID-VISTA trials for aflibercept, and numerous studies with level 2 and 3 evidence for bevacizumab demonstrated that almost 40% of patients gained 15 letters or more on Snellen eye charts at two years of follow-up [4–8]. Although a significant proportion of patients had visual and anatomic improvement in prospective multicenter studies with regular treatment and follow-up schedules, a considerable amount of patients showed poor response to current anti-VEGF treatment. Hence, it is logical to switch anti-VEGF agents between each other if the previous treatment is not sufficient to resolve macular edema. However, few studies have assessed the results of switching anti-VEGF therapies in patients with poor response to DME [9–12]. In light of these findings, there is still a question that remains to be answered regarding whether macular edema resolves when previous treatment is continued. To date, there are limited data about switching anti-VEGF agents regarding their effectiveness in DME. The aim of this study was to address the outcomes of aflibercept use in patients who did not respond to previous ranibizumab treatment. Therefore, the visual and anatomic outcomes of switching therapy from ranibizumab to aflibercept were compared with those of patients treated with ranibizumab only in persistent/nonresolving macular edema secondary to diabetes.

## 2. Methods

In this retrospective, observational, comparative case series, data were collected from the records of sequential patients who were followed up for DME. To identify eligible patients who were both treated with ranibizumab injections (0.5 mg/0.05 mL) continuously and previously treated with ranibizumab and were subsequently switched to aflibercept (2 mg/0.05 mL), electronic medical records of patients with DME between August 2015 and May 2017 were reviewed. Written informed consent was obtained from all patients before the injections, and the protocol of the study adhered to the tenets of the Declaration of Helsinki.

To be included in the study, each patient was required to meet all of the following criteria: patients with type 2 diabetes aged ≥18 years, center-involving DME (central macular thickness (CMT) ≥ 350 *μ*m), and best corrected visual acuity (BCVA) of ≥20/400. Patients were excluded if they had any of the following treatments within 6 months prior to study entry: intravitreal or sub-Tenon's injections of steroids, intravitreal dexamethasone implant, intravitreal anti-VEGF injections, focal/grid macular laser photocoagulation, panretinal photocoagulation, cataract surgery, or pars plana vitrectomy. Patients who had macular edema secondary to a cause other than diabetes or any concomitant ocular pathologies aside from diabetic retinopathy or vitreoretinal surface disorders were also excluded.

Afterwards, the patients (*n* = 43) were divided into two groups: the switch group (*n* = 20) consisted of patients who demonstrated poor response or an increase in CMT after the last three monthly ranibizumab injections following former ranibizumab treatment and then switched to aflibercept and the ranibizumab group (*n* = 23) comprised patients who demonstrated a poor response (decrease in CMT < 10%) after the last three monthly ranibizumab injections following former ranibizumab treatment and then continued to receive ranibizumab injections.

In the presence of persisting subretinal or intraretinal fluid, treatment with ranibizumab or aflibercept was continued using an as-needed regimen until no improvement in CMT was seen.

The decision to treat using an as-needed regimen, which followed an optical coherence tomography- (OCT-) guided treatment protocol, was made by a retina specialist. If no center-involved macular edema was seen, monthly monitoring visits were arranged and further injections of ranibizumab or aflibercept were withheld. In case of newly formed or persistent macular edema or increase in CMT ≥ 50 *μ*m compared with the previous visit, retreatment with either intravitreal ranibizumab or aflibercept was applied.

At each visit, a complete ophthalmologic examination including measurement of BCVA using Snellen charts, slit-lamp biomicroscopy, intraocular pressure measurement using applanation tonometry, and dilated biomicroscopic fundus examination was conducted and OCT imaging using a SPECTRALIS OCT (SPECTRALIS; Heidelberg Engineering, Heidelberg, Germany) was performed. Data were collected at presentation (preinjection), at the intermediary visit (preswitch visit in the switch group and 4–6 weeks after the last injection of three monthly ranibizumab injections in the ranibizumab group), and at the last visit (at the end of the follow-up period). Only data of patients who completed a minimum 6-month follow-up period after the intermediary visit were collected for analysis.

CMT, which is defined as the mean thickness of the neurosensory retina in the central 1 mm diameter, was computed through OCT mapping software provided by the device. OCT characteristics of DME were classified as cystoid macular edema (CME), serous retinal detachment (SRD), and sponge-like retinal swelling [13]. CME associated with or without sponge-like retinal swelling was classified as CME. The presence of disorganization of inner retinal layers (DRIL) and disruption of the ellipsoid zone (EZ) (formerly termed inner segment/outer segment photoreceptor junction) were evaluated on the central B scan which was identified as the central scan passing through the central foveal area on the infrared image. DRIL was defined as any irregularity obscuring the well-delineated boundaries between the inner retinal layers (the ganglion cell-inner plexiform layer complex, inner nuclear layer, and outer plexiform layer). Foveal 1 mm zone was evaluated for the presence of DRIL and disruption of EZ. If ≥50% of the central foveal 1 mm zone was affected by DRIL, then DRIL was considered as present according to a previous study [14]. If EZ was disrupted within the 1 mm foveal area, EZ was graded as not intact [15]. B scans were evaluated by two independent specialists (Ali Demircan and Zeynep Alkin). The observed agreement between the 2 graders was 92.7%. All disagreement scans were resolved by mutual agreement.

The demographic features of patients at baseline, BCVA and CMT values obtained at all visits, and the mean number of anti-VEGF injections at the first and last visits were recorded. The mean changes in CMT and BCVA from baseline at the last visit were the primary outcomes and were used to compare the efficacy of both treatments. The percentage of patients who gained ≥1 line in BCVA, with CMT < 350 *μ*m at the last visit, and with ≥10% reduction in CMT were secondary outcomes.

### 2.1. Statistical Analysis

Data were analyzed using SPSS 22.0 program (SPSS Chicago, Illinois, USA). Snellen BCVA was converted into logarithm of the minimal angle of resolution (logMAR) for statistical analysis. Continuous variables are expressed as mean ± standard deviation (SD). Categorical variables are expressed as numbers (*n*) and percentages (%). The distribution of the variables was measured using the Kolmogorov–Smirnov test. The Mann–Whitney *U* test was used for the analysis of independent quantitative data. The Wilcoxon test was used for the analysis of dependent quantitative data. The chi-square test was used to analyze independent qualitative data, and Fisher's exact test was used when chi-square test conditions were not met. Spearman's correlation analysis was used for correlation analyses.

## 3. Results

A total of 43 eyes of 43 patients were included; these comprised both patients who switched from ranibizumab to aflibercept (switch group, *n* = 20) and those treated with ranibizumab only (ranibizumab group, *n* = 23). The mean age was 62.1 ± 7.5 years in the switch group and 63.4 ± 6.5 years in the ranibizumab group. No significant difference was found between the groups (*p* = 0.37). The demographics and clinical characteristics of the patients in both groups are shown in Table 1.

The mean BCVA (logMAR) in the switch and ranibizumab groups was 0.67 ± 0.38 (range: 1.3–0.2) and 0.73 ± 0.34 (range: 1.3–0.15), respectively, at presentation. No statistically significant difference was found between the groups (*p* = 0.55). In the switch group, the mean BCVA (logMAR) improved from 0.68 ± 0.40 at the intermediary visit to 0.58 ± 0.38 at the last visit. Compared with the intermediary visit, there was no statistically significant improvement at the last visit (*p* = 0.08). In the ranibizumab group, the mean BCVA (logMAR) improved from 0.71 ± 0.37 at the intermediary visit to 0.67 ± 0.37 at the last visit; no significant difference was found at the last visit compared with the intermediary visit (*p* = 0.12).

**Figure 1:**
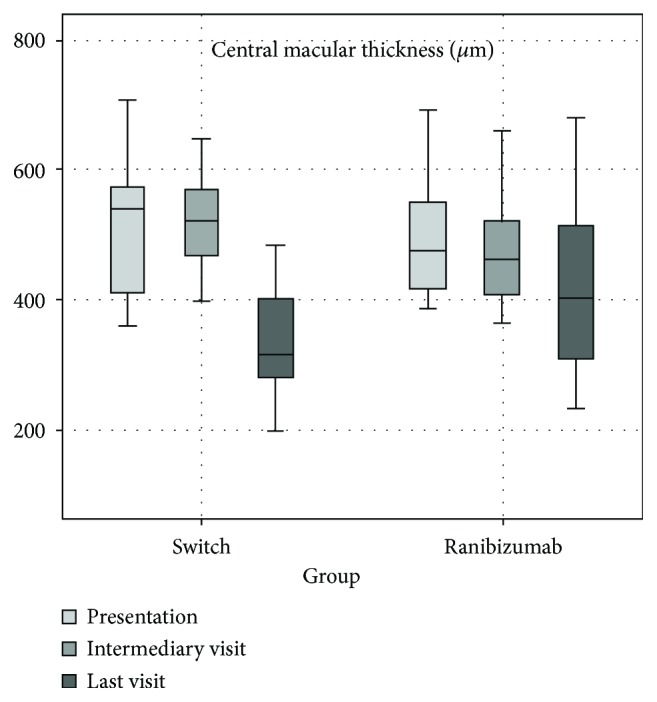


The changes in the mean CMT of the two groups are shown in Figure 1. The mean CMT in the switch and ranibizumab groups was 506.9 ± 102.2 *μ*m (range: 360–707 *μ*m) and 487.3 ± 82.6 *μ*m (range: 387–692 *μ*m) at presentation and 530.7 ± 91.8 *μ*m and 473.5 ± 78.4 *μ*m at the intermediary visit. No statistically significant difference was found between the groups (*p* = 0.53, *p* = 0.07, resp.).

The mean CMT decreased from 530.7 ± 91.8 *μ*m and 473.5 ± 78.4 *μ*m at the intermediary visit to 342.1 ± 87.5 *μ*m and 413.2 ± 123.8 *μ*m at the last visit in the switch and ranibizumab groups, respectively. Compared with the intermediary visit, there was a significant decrease at the last visit in the switch and ranibizumab groups (*p* < 0.001 and *p* = 0.03 resp.). The mean CMT decreased after the intermediary visit by 188.6 ± 120.5 *μ*m in the switch group and by 60.3 ± 117.1 *μ*m in the ranibizumab group. A significant difference was found in CMT reduction between the switch group and the ranibizumab group (*p* = 0.003).

At the last visit, 5 of 20 eyes (25%) in the switch group and 4 of 23 eyes (17.3%) in the ranibizumab group showed a ≥1 line improvement in BCVA. The number of eyes with ≥10% reduction in CMT at the last visit was 18 of 20 eyes (90%) in the switch group and 11 of 23 eyes (47.8%) in the ranibizumab group. There were 12 of 20 eyes (60%) in the switch group and 7 of 23 eyes (34.7%) in the ranibizumab group in which CMT was <350 *μ*m at the last visit.

At the intermediary visit, 20 of the 20 eyes (100%) in the switch group and 23 of the 23 eyes (100%) in the ranibizumab group had CME on OCT. SRD was present in 8 eyes (40%) in the switch group and 5 eyes (21.7%) in the ranibizumab group. Eight eyes (40%) in the switch group and 6 eyes (26%) in the ranibizumab group had the presence of DRIL. EZ disruption was present in 9 eyes (45%) in the switch group and 7 eyes (30.4%) in the ranibizumab group.

The mean number of ranibizumab injections was 5.3 ± 1.2 (range: 4–9) in the switch group and 5.5 ± 0.9 (range: 4–7) in the ranibizumab group before the intermediary visit in a mean period of 12 months. No statistically significant difference was found between the groups (*p* = 0.64). Eyes in the switch and ranibizumab groups received a mean number of 3.5 ± 0.7 (range: 3–5) and 3.7 ± 0.6 (range: 3–5) injections from the intermediary visit and the last visit, respectively, with a mean duration of 6.7 ± 0.8 months. There was no significant difference between the switch group and the ranibizumab group in the mean number of injections after the intermediary visit (*p* = 0.32).

## 4. Discussion

Vascular endothelial growth factor is an important mediator in the pathogenesis of DME. Intravitreal injections of anti-VEGFs have been established as the main treatment of DME in the last few years. In spite of regular treatment, there are a proportion of patients who incompletely respond to anti-VEGF agents. The Diabetic Retinopathy Clinical Research Network (DRCRnet) Protocol I showed that 52% of patients treated with ranibizumab failed to achieve ≥2 line improvement in BCVA and that 40% had no resolution of retinal thickening at the second year [16]. When treating DME with anti-VEGF agents, the physician has the option of trying other anti-VEGFs or corticosteroids in patients with poor response. Although there are no large randomized prospective clinical trials comparing treatment regimens for refractory DME, several smaller uncontrolled studies demonstrated visual and/or morphologic improvement after switching patients who showed poor response from aflibercept to ranibizumab injections [9–12].

Lim et al. reported visual and morphologic improvements after switching to aflibercept in 21 eyes of 19 patients with DME who had a poor response to multiple bevacizumab/ranibizumab injections [11]. A study by Bahrami et al. similarly demonstrated the beneficial effect of aflibercept on both visual improvement as well as morphologic improvement in patients with DME who had poor response to previous bevacizumab injections [17]. Wood et al. showed only morphologic improvement with aflibercept in patients with poor response to ranibizumab and/or bevacizumab injections in their prospective study [18]. However, the majority of patients (11 of 14) in their study were evaluated after only one aflibercept injection. Rahimy et al. also demonstrated only a morphologic response to aflibercept injections after previous bevacizumab/or ranibizumab therapy, and they explained this result by irreversible functional damage caused by long-standing DME [19]. Switching to aflibercept resulted in some anatomic improvement in the majority of patients in all studies.

In our study, both ranibizumab and aflibercept treatments provided only morphologic improvement in patients who have poor response to previous ranibizumab treatment. A greater decrease in macular thickness in the switch group than in the ranibizumab group in the current study might be explained by the blocking of all isoforms of VEGF-A, VEGF-B, and PlGF with aflibercept in contrast to inactivation of only VEGF-A with ranibizumab. Some studies showed that PlGF may have a place in the pathogenesis of DME. Increasing intravitreal concentrations of PlGF has been associated with progressively advancing degrees of diabetic retinopathy [20–23]. Blockade of this protein might play a role in such patients. Moreover, the greater improvement in macular morphology with aflibercept might be related to patients' inherent characteristics rather than features of aflibercept. In addition to all these possible explanations, patients treated with repetitive ranibizumab/bevacizumab injections may demonstrate tachyphylaxis or a diminished therapeutic response to these agents over time as suggested in a great number of studies [24, 25]. Additionally, there was a trend towards greater visual acuity improvement after switching to aflibercept, but it was not statistically significant. The discrepancy between morphologic and functional outcomes may be explained by irreversible functional damage caused by long-standing DME. Switching to intravitreal steroids with good functional and morphologic outcomes after ranibizumab failure in DME treatment has been shown in previous studies [26]. A switch to another pharmaceutical class such as corticosteroids is a logical option in case of failure of other therapies in DME.

All of the previous studies only reported outcomes of patients with a poor response to bevacizumab/ranibizumab who switched to aflibercept and had no comparison between the outcomes of switched patients and those of patients who continued with previous anti-VEGF treatment. It is not clear whether the visual and/or anatomic recovery in these patients originated from the new intravitreal anti-VEGF agent or from the total number of anti-VEGF injections applied because it was demonstrated that there was a delayed responder group treated with ranibizumab that showed some visual and anatomic improvement when treatment was continued with further ranibizumab injections.

The major limitations of this study were the relatively small sample size and short follow-up time as well as its retrospective design. Further prospective and randomized studies with larger sample sizes and longer duration are needed to evaluate the effectiveness of aflibercept injections in the visual and morphologic improvements following changing previous treatment in persistent DME.

In the current study, we compared a switch group that comprised patients who switched to aflibercept after showing a poor response to previous ranibizumab treatment with a ranibizumab group composed of patients who continued with ranibizumab injections despite the presence of poor response to this treatment. To the best of our knowledge, this is the first study in the literature to compare these treatments in persistent DME.

In conclusion, the results of our study showed that switching therapy from intravitreal ranibizumab to aflibercept in persistent DME provided only morphologic improvement. The discrepancy between morphologic and functional outcomes may be explained by irreversible functional damage caused by long-standing DME.

## Figures and Tables

**Table 1:** Demographics and number of ranibizumab injections in both groups.

	Switch group *n* = 20	Ranibizumab group *n* = 23	*p*
Age (years)			0.37
Mean (±SD)	62.1 ± 7.5	63.4 ± 6.5	
Median (min–max)	60 (50–76)	64 (53–72)	
Gender			0.09
Male	9 (45%)	13 (56.5%)	
Female	11 (55%)	10 (43.4%)	
Number of ranibizumab injections before intermediary visit			0.64
Mean (±SD)	5.3 ± 1.2	5.5 ± 0.9	
Median (min–max)	5 (3–5)	5 (3–5)	

*n*: number; SD: standard deviation.
